# Data supporting the cardiac mitochondria calcium handling in female normotensive and spontaneously hypertensive rats

**DOI:** 10.1016/j.dib.2016.02.056

**Published:** 2016-02-27

**Authors:** Alejandro Ciocci Pardo, Gustavo J. Rinaldi, Susana M. Mosca

**Affiliations:** Centro de Investigaciones Cardiovasculares “Dr. Horacio E. Cingolani”, CCT-CONICET, Facultad de Ciencias Médicas, Universidad Nacional de La Plata, Argentina

**Keywords:** Cardiac mitochondria, Calcium, Female WKY, Female SHR

## Abstract

In association with the published article “Mitochondrial calcium handling in normotensive and spontaneously hypertensive rats: correlation with systolic blood pressure levels” [1], this data article contains information about calcium handling of cardiac mitochondria isolated from female of both rats strains (WKY and SHR). Dataset of mitochondrial permeability transition pore (mPTP) resistance to opening Ca^2+^-mediated, Ca^2+^ retention capacity (CRC), time constants and mitochondrial membrane potential (ΔΨm) are showed.

## 1 **Specifications Table**

**Table:** 

Subject area	*Cardiovascular*
More specific subject area	*Mitochondria*
Type of data	*Table and figures*
How data was acquired	*Spectrofluoremeter Hitachi F4500*
Data format	*Mean±SE*
Experimental factors	*Isolated mitochondria from hearts of female normotensive (WKY) and spontaneously hypertensive rats (SHR).*
Experimental features	mPTP resistance to opening Ca^2+^-mediated, Ca^2+^-retention capacity, time constants and mitochondrial membrane potential were tested to assess the calcium handling.
Data source location	*La Plata (Buenos Aires), Argentina*
Data accessibility	*Data are within this article*

## 2 **Value of the data**

• The data provide information about calcium handling of cardiac mitochondria obtained from female normotensive and hypertensive rats.
• The data can be compared to data obtained in male rats of both strains [1].
• The data can be used in the development of further experiments to analyze the influence of sex.

## 3 Data

**Fig. 1:**
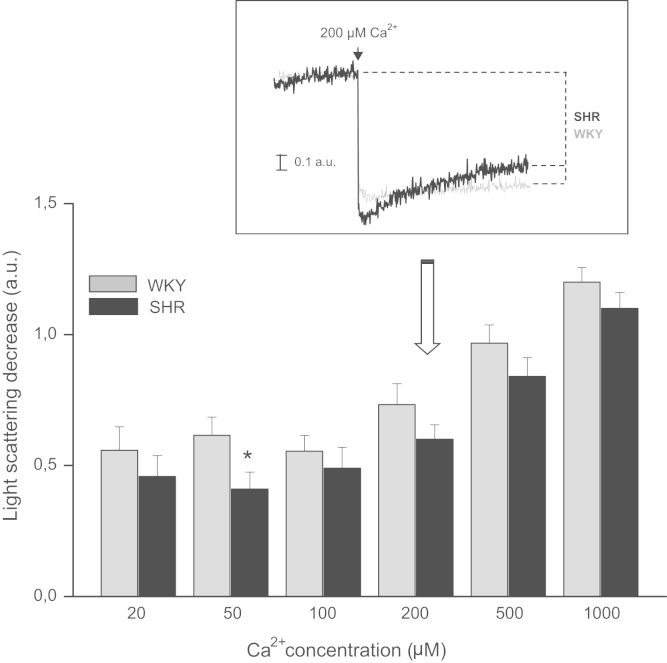
Light scattering decrease (LSD) in arbitrary units (a.u.) after the addition of different Ca^2+^ concentrations (20, 50, 100, 200, 500 and 1000 µM) to samples of mitochondria isolated from female WKY and SHR hearts. Inset: typical trace of light scattering for SHR and WKY at Ca^2+^ 200 µM. Note that only at Ca^2+^ 50 µM LSD was significantly lesser in SHR than WKY. ^*^p<0.05.

**Fig. 2:**
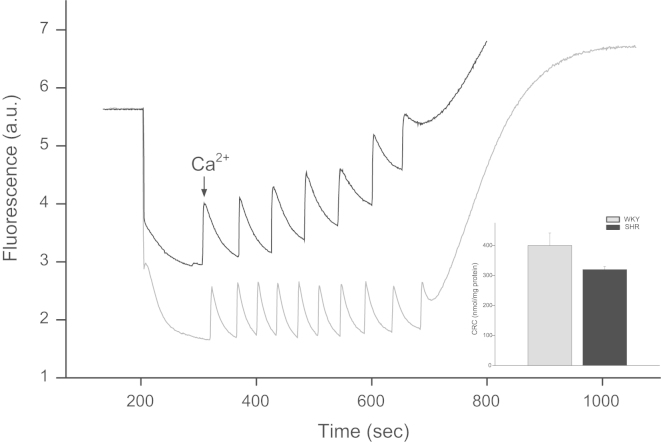
Changes of **c**alcium green fluorescence after Ca^2+^ addition in samples of mitochondria from female WKY and SHR hearts. Inset: Ca^2+^ retention capacity (CRC) in both rats strains. Note that no statistically significant difference between WKY and SHR was detected.

**Fig. 3:**
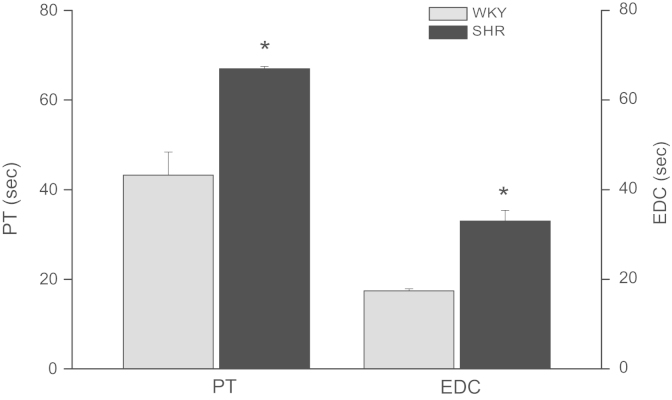
Pulse time (PT) and exponential decay constant (EDC) of WKY and SHR female. Both parameters were greater in SHR than WKY. *p<0.05.

**Fig. 4:**
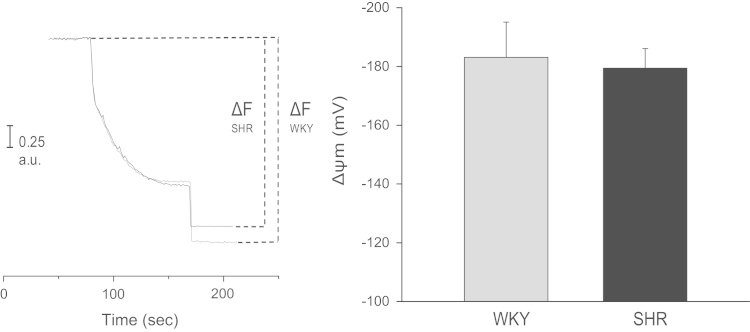
Typical traces of fluorescence changes and mean values of mitochondrial membrane potential (ΔΨm) of samples obtained from female WKY and SHR hearts. ΔΨm was similar in both rats strains.

Systolic blood pressure (SBP), body weight (BW), heart weight (HW) and hypertrophy index (HI, calculated as HW and BW ratio) of female WKY and SHR are displayed in Table 1. The mPTP resistance to opening, measured as light scattering decrease, after different Ca^2+^ concentrations in samples derived from female of both rats strains was depicted in Fig. 1. Ca^2+^ retention capacity (CRC), the time of each pulse (PT) and exponential decay constant (EDC), obtained after addition of successive pulses of 10 µM Ca^2+^ are shown in Fig. 2, Fig. 3. Rhodamine fluorescence changes used for the membrane potential (ΔΨm) determination in cardiac mitochondria of WKY and SHR hearts appear in Fig. 4.

## 4 Experimental design, materials and methods

### 4.1 Animal model

We used female normotensive (Wistar Kyoto, WKY) and spontaneously hypertensive rats (SHR) of 5 months of age. All procedures followed during this investigation conform to the Guide for the Care and Use of Laboratory Animals published by the US National Institutes of Health (NIH Publication no. 85-23, revised 1996) and to the guidelines laid down by the Animal Welfare Committee of La Plata School of Medicine.

### 4.2 Characteristics of WKY and SHR

Values of SBP, BW, HW and HI in WKY and SHR are shown in Table 1.

### 4.3 Isolation of rat heart mitochondria

Mitochondria were obtained by differential centrifugation following the method described by Mela and Seitz [2].

### 4.4 Mitochondrial permeability transition pore (mPTP) resistance to opening Ca^2+^-mediated

The mPTP resistance to opening was assessed by addition of different Ca^2+^ concentrations to samples of 0.3 mg/mL of isolated mitochondria from hearts of WKY and SHR female. The changes were observed as decreases of light scattering and followed using a temperature-controlled Hitachi F4500 spectrofluorometer operating with continuous stirring at excitation and emission wavelengths of 520 nm [3]. Light scattering decrease (LSD) was calculated for each sample by taking the difference of scattered light between before and after the addition of CaCl_2_ (Fig. 1).

### 4.5 Calcium retention capacity (CRC)

CRC (Fig. 2) was defined as the amount of Ca^2+^ required triggering a massive Ca^2+^ release by isolated cardiac mitochondria [4]. Briefly, successive pulses of 10 µM Ca^2+^ were added to samples of 0.3 mg/mL of isolated mitochondria of hearts from WKY and SHR. After sufficient Ca^2+^ loading, extramitochondrial Ca^2+^ concentration abruptly increased, which was recorded by fluorescence changes of Calcium green-5N with excitation and emission wavelengths set at 506 and 532 nm, respectively. Pulse time (PT, s) and exponential decay constant (EDC) of Ca^2+^ influx to mitochondria were also measured (Fig. 3).

### 4.6 Mitochondrial membrane potential (ΔΨm)

Mitochondrial potential changes were evaluated by measuring rhodamine-123 (RH-123) fluorescence quenching under the buffer described above containing RH-123 0.1 μM [5] and using 0.1 mg/mL of isolated mitochondria. ΔΨm was calculated following the instructions previously detailed by Scaduto and Grotyohann [6], using the Nernst–Guggenheim equation (Fig. 4).

## Figures and Tables

**Table 1:** Values of SBP, BW, HW and HI for female WKY and SHR.

	**WKY**	**SHR**
**SBP (mmHg)**	118±7	160±5⁎
**BW (g)**	217±2	218±7
**HW (mg)**	595±30	732±40⁎
**HI**	2.77±0.16	3.24±0.14⁎

⁎ *p*<0.05 SHR vs WKY.
